# Targeting the Acidic Tumor Microenvironment: Unexpected Pro-Neoplastic Effects of Oral NaHCO_3_ Therapy in Murine Breast Tissue

**DOI:** 10.3390/cancers12040891

**Published:** 2020-04-06

**Authors:** Ninna C. S. Voss, Thomas Dreyer, Mikkel B. Henningsen, Pernille Vahl, Bent Honoré, Ebbe Boedtkjer

**Affiliations:** 1 Department of Biomedicine, Aarhus University, DK-8000 Aarhus, Denmark; ninnschm@rm.dk (N.C.S.V.); thomas@clin.au.dk (T.D.); bakmikkel@hotmail.com (M.B.H.); bh@biomed.au.dk (B.H.); 2 Department of Surgery, Regionshospitalet Randers, DK-8930 Randers, Denmark; 3 Department of Pathology, Aarhus University Hospital, DK-8200 Aarhus, Denmark; pernvahl@rm.dk

**Keywords:** acidosis, buffer therapy, bicarbonate, mass spectrometry, pH, proteomics

## Abstract

The acidic tumor microenvironment modifies malignant cell behavior. Here, we study consequences of the microenvironment in breast carcinomas. Beginning at carcinogen-based breast cancer induction, we supply either regular or NaHCO_3_-containing drinking water to female C57BL/6j mice. We evaluate urine and blood acid-base status, tumor metabolism (microdialysis sampling), and tumor pH (pH-sensitive microelectrodes) in vivo. Based on freshly isolated epithelial organoids from breast carcinomas and normal breast tissue, we assess protein expression (immunoblotting, mass spectrometry), intracellular pH (fluorescence microscopy), and cell proliferation (bromodeoxyuridine incorporation). Oral NaHCO_3_ therapy increases breast tumor pH in vivo from 6.68 ± 0.04 to 7.04 ± 0.09 and intracellular pH in breast epithelial organoids by ~0.15. Breast tumors develop with median latency of 85.5 ± 8.2 days in NaHCO_3_-treated mice vs. 82 ± 7.5 days in control mice. Oral NaHCO_3_ therapy does not affect tumor growth, histopathology or glycolytic metabolism. The capacity for cellular net acid extrusion is increased in NaHCO_3_-treated mice and correlates negatively with breast tumor latency. Oral NaHCO_3_ therapy elevates proliferative activity in organoids from breast carcinomas. Changes in protein expression patterns—observed by high-throughput proteomics analyses—between cancer and normal breast tissue and in response to oral NaHCO_3_ therapy reveal complex influences on metabolism, cytoskeleton, cell-cell and cell-matrix interaction, and cell signaling pathways. We conclude that oral NaHCO_3_ therapy neutralizes the microenvironment of breast carcinomas, elevates the cellular net acid extrusion capacity, and accelerates proliferation without net effect on breast cancer development or tumor growth. We demonstrate unexpected pro-neoplastic consequences of oral NaHCO_3_ therapy that in breast tissue cancel out previously reported anti-neoplastic effects.

## 1. Introduction

The acidic tumor microenvironment is a hallmark of solid cancer tissue with multifaceted consequences for cancer cells, adjacent normal cells, and their interactions [1,2]. Enhanced metabolic acid production in cancer tissue is a consequence of accelerated glycolysis and/or oxidative phosphorylation that provide energy and chemical intermediates for cancer cell proliferation [3]. Cancer cells export the increased acid load to the extracellular space via acid-base transporters in their cell membranes [4,5,6]. Inadequate perfusion further amplifies local interstitial accumulation of acid [7]. The electroneutral Na^+^, HCO_3_^–^-cotransporter NBCn1 (Slc4a7) is upregulated in human and murine breast cancer tissue compared to normal breast tissue; and acting in parallel with the Na^+^/H^+^-exchanger isoform 1 (NHE1, Slc9a1), NBCn1 is the predominant path for net acid extrusion from breast cancer cells [8,9,10,11]. Cellular elimination of lactate and H^+^ from glycolytic metabolism can also occur via monocarboxylate transporters [12,13]. Acid-base transport processes across membranes, within the cytoplasm, and through the tortuous extracellular space can be accelerated by carbonic anhydrases that catalyze the reaction CO_2_ + OH^−^ ⇄ HCO_3_^−^ [14,15,16]. The functional consequences of carbonic anhydrases have hitherto been studied mostly in model systems and need to be confirmed in breast cancer tissue. Despite the high metabolic rate and low extracellular pH (pH_o_), efficient net acid extrusion maintains the cytoplasm of breast cancer cells more alkaline than normal breast epithelial cells under the same conditions [8,9]. The characteristic compartmentalization of acidity in solid cancer tissue—with very low pH_o_ and relatively high intracellular pH (pH_i_)—is an early event in carcinogenesis and a promising therapeutic target [4,17,18].

Local extracellular acidity is believed to promote cancer development and progression but it is a point of contention whether pH_o_ within tumors can reach levels so low that it restricts functions of acid-adapted cancer cells [17]. Advancement through specific cell cycle checkpoints depends on transient increases in pH_i_ [19,20], and acidosis typically inhibits cell cycle progression and hence proliferation of cultured cancer cells [21]. However, opposing facilitatory roles of acidosis on proliferation are possible as extracellular H^+^ acting directly on H^+^-sensing receptors or H^+^-activated ion channels may elicit signals that favor proliferation [17]. Despite these uncertainties, it is well-accepted that cancer cells show improved resistance to acidosis compared to normal cells [8] and that the evolutionary selection pressure of the acidic microenvironment can favor more malignant phenotypes [22]. The overall consequences of the tumor microenvironment depend on the degree of acidification; and adaptive processes initiated by tumor acidosis likely enhance cancer cell function particularly if they later encounter less acidic environments, for instance, during invasion [17].

Acidosis can influence cellular functions by modulation of enzymatic activities, and particularly the acid-sensitivity of enzymes in the glycolytic pathway has attracted attention [23]. Cancer cells respond to sustained acidosis by reshaping their metabolic phenotype and over time shift towards glutamine metabolism and fatty acid oxidation [24,25,26]. Acidosis also has cancer type-dependent effects on autophagy as illustrated by the stimulatory effect of low pH_o_ in melanoma cell lines yet inhibitory effect in breast cancer cells [27,28]. Cancer-promoting effects of low pH_o_ include degradation of extracellular matrix—through acid-mediated activation of matrix metalloproteinases, cathepsins, lysosomal proteases, and hyaluronidase [29,30,31,32]—and induction of cell death in surrounding normal cells [33]. The low pH_o_ of the tumor microenvironment also purportedly inhibits immune cell functions [17,34]. Together, these processes facilitate tumor expansion and metastasis and are essential for the prognosis of cancer patients.

Ingestion of base, through dietary intake or buffer therapy, has been proposed to reduce the risk of cancer development and metastasis [35,36]. Although oral administration of NaHCO_3_—in order to neutralize the tumor microenvironment—does not affect primary breast tumor growth in murine xenograft models [32], it lowers the number of metastases and prevents recurrence [32,37]. As xenograft models involve in vivo implantation of already transformed malignant cells, they are not well suited for studying processes whereby normal cells transform into cancer cells. Supporting a role of pH during carcinogenesis, oral NaHCO_3_ therapy impairs primary tumor growth in the transgenic adenocarcinoma of the mouse prostate (TRAMP) model [38], and knockout of NBCn1 delays carcinogen- and ErbB2-induced breast cancer development and decelerates tumor growth [9,10]. Evidence from human intervention and observational studies is still insufficient to support or exclude that an alkaline diet can be used for prevention or treatment of cancer [39]. Initial studies, showing promising effects of alkaline therapy on renal function in patients with chronic kidney disease, suggest that alkaline supplements can be administered to humans without unacceptable adverse effects [40].

In the current study, we explore consequences of oral NaHCO_3_ therapy during breast carcinogenesis. Contrary to our expectation, the elevated pH_o_ of the tumor microenvironment in response to oral NaHCO_3_ therapy shows no overall net effect on breast tumor-free survival or primary tumor growth. We reveal unexpected pro-neoplastic effects of oral NaHCO_3_ therapy in breast tissue—including increased capacity for cellular net acid extrusion and accelerated in vitro proliferation—that likely cancel out previously reported benefits of oral buffer therapy. Consistent with this model of mixed pro- and anti-neoplastic consequences of tumor acidosis, we show—based on high-throughput proteomics analyses—that oral NaHCO_3_ therapy differentially influences protein expression patterns related to cell signaling, metabolism, cytoskeleton, and cell-cell and cell-matrix interaction during breast carcinogenesis.

## 2. Results

### 2.1. Oral NaHCO_3_ Therapy Alkalinizes Tumors, Urine, and Arterial Blood

**Figure 1:**
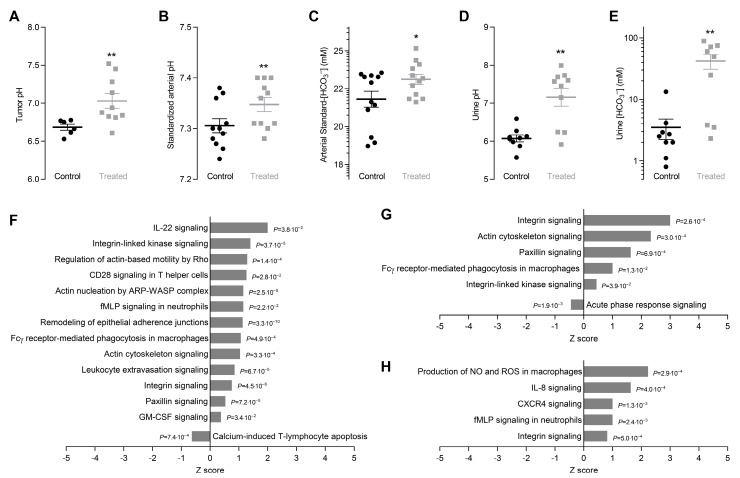
Oral NaHCO_3_ therapy alkalinizes breast tumors and increases [HCO_3_^–^] and pH of arterial blood and urine. It also leads to marked protein expression changes related to immune function, cytoskeleton, and cell-cell and cell-matrix interaction. (**A–C**)**.** Tumor pH (**A**) along with pH (**B**) and standard-[HCO_3_^–^] (**C**) of arterial blood from NaHCO_3_-treated mice and control mice (*n* = 6–11) mechanically ventilated to normocapnia (expiratory end-tidal CO_2_ fraction of 3.8%). (**D,E**)**.** Urine pH (**D**) and [HCO_3_^–^] (**E**) of NaHCO_3_-treated mice and control mice (*n* = 9–10). (**F–H**)**.** Protein expression changes related to immune function, cytoskeleton, and cell-cell and cell-matrix interaction. We compare breast cancer tissue vs. normal breast tissue from control mice (**F**), breast cancer tissue from NaHCO_3_-treated mice vs. control mice (**G**), and normal breast tissue from NaHCO_3_-treated mice vs. control mice (**H**). Data in panel C were log-transformed in order to improve normal distribution. Data in panel A were compared by unpaired two-tailed *t*-test with Welch’s correction for unequal variance, data in panel B through D were compared by unpaired two-tailed Student’s *t*-tests, and data in panel E were compared by the non-parametric Mann-Whitney test. *p*-values in panel F through H were calculated based on Fisher’s right-tailed exact test. *p < 0.05, **p < 0.01 vs. Control.

The microenvironment of breast cancer tissue in vivo is highly acidic reaching an average pH of 6.68 ± 0.04 (Figure 1A), which is consistent with previous reports [41]. Chronic oral administration of NaHCO_3_ elevates tumor pH by 0.35 ± 0.13 (Figure 1A).

Sustained oral NaHCO_3_ therapy also elevates pH (Figure 1B) and standard-[HCO_3_^−^] (Figure 1C) of arterial blood although the alkalinizing effect is considerably smaller than for tumors. We performed recordings on and collected blood samples from mice mechanically ventilated to normocapnia in order to avoid stress-induced hyperventilation or anesthesia-induced hypoventilation. Accordingly, pCO_2_ of arterial blood was similar (*p* = 0.78, unpaired two-tailed Student’s *t*-test) for NaHCO_3_-treated mice (45.10 ± 2.23 mmHg) and control mice (47.85 ± 2.31 mmHg). At steady-state, intake and output of NaHCO_3_ must balance, and we indeed document dramatic increases in urinary pH (Figure 1D) and [HCO_3_^–^] (Figure 1E) during oral NaHCO_3_ therapy.

### 2.2. Oral NaHCO_3_ Therapy Modifies Protein Expression Related to Cytoskeleton, Cell-Cell and Cell-Matrix Interaction, and Immune Function

We identify marked differences in protein expression patterns related to cell-cell and cell-matrix interactions, cytoskeletal dynamics, and immune-related functions between organoids freshly isolated from breast cancer tissue and matched normal breast tissue (Figure 1F and Figure S1A). These effects are anticipated based on the changes in cytoskeleton, extracellular matrix, and cell-cell interactions that occur during cancer development [17]. Our data suggest that oral NaHCO_3_ therapy amplifies or perturbs carcinogen-induced protein expression changes in breast cancer tissue (Figure 1G and Figure S1B) and even cause related changes in matched macroscopically normal breast tissue (Figure 1H and Figure S1C). These data imply that acidosis of the breast cancer microenvironment in some respects limits the cellular alterations taking place during carcinogenesis.

Acidosis of the tumor microenvironment purportedly attenuates immune-mediated anti-neoplastic responses in cancer tissue [42]. In congruence, we find that signaling pathways of particular relevance for macrophage and neutrophil function are perturbed in the breast tissue of NaHCO_3_-treated mice, including chemokine signaling downstream of interleukin (IL)-8, *N*-formyl-methionyl-leucyl-phenylalanine (fMLP), and the C-X-C motif chemokine receptor (CXCR)4, Fcγ-receptor-mediated phagocytosis, and macrophage production of NO and reactive oxygen species (ROS) (Figure 1G,H). The detailed mechanisms of macrophage infiltration and function in solid cancer tissue are not well understood but subtype-dependent differences have been described with M1-like macrophages predominantly suppressing and M2-like macrophages predominantly promoting tumor growth [43]. Variable influences [44,45,46,47,48] of the immune-relevant signaling pathways that we find perturbed by oral NaHCO_3_ therapy (Figure 1G,H) likely reflect the complexity of the interaction between immune cells, cancer cells, and the tumor microenvironment. Overall, our findings support that interventions, such as oral NaHCO_3_ therapy, to neutralize the acidity of the tumor microenvironment modify protein expression patterns (Figure 1G,H and Figure S1B,C) that may otherwise contribute to immune evasion in solid cancer tissue.

### 2.3. Oral NaHCO_3_ Therapy does not Affect Tumor Burden, Tumor-Free Survival, or Histopathology

**Figure 2:**
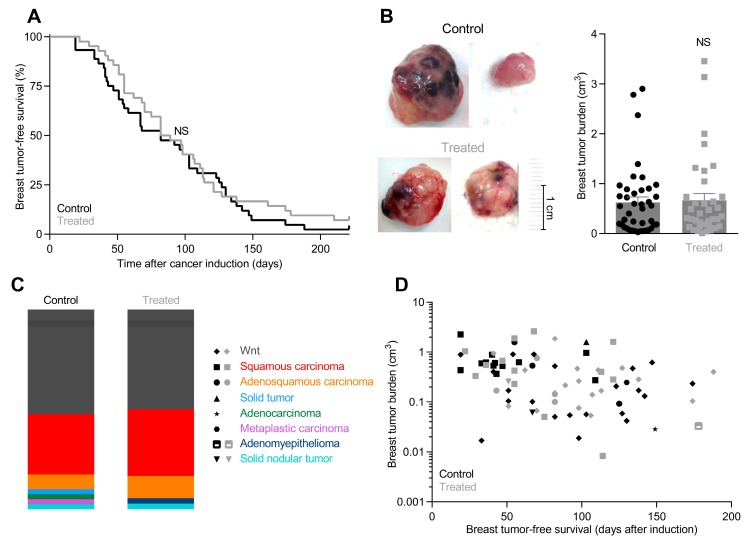
Tumor-free survival, tumor burden, and breast tumor histopathology are similar in NaHCO_3_-treated mice and control mice. (**A**). Tumor-free survival of NaHCO_3_-treated mice and control mice after carcinogen-based breast cancer induction. Median tumor-free survival of NaHCO_3_-treated mice was 85.5 ± 8.2 days (*n* = 42) compared to 82.0 ± 7.5 days for control mice (*n* = 45). Data were compared by Gehan-Breslow-Wilcoxon test. (**B**). Tumor burden was similar in NaHCO_3_-treated mice and control mice (*n* = 37–41) two weeks after first tumor detection. Tumors were typically 3–4 mm at first detection consistent with previous reports [49]. Data were log-transformed in order to improve normal distribution and then compared by unpaired two-tailed Student’s *t*-test. (**C**). Distribution of breast tumors from NaHCO_3_-treated mice and control mice (*n* = 37–41) between histopathological subtypes. (**D**). Plot of matched data for histopathology, tumor burden, and tumor latency of breast tumors from NaHCO_3_-treated mice and control mice (*n* = 37–41). NS: Not significantly different vs. Control.

After carcinogen-based induction, breast tumors develop with a median latency of 85.5 ± 8.2 days in NaHCO_3_-treated mice compared to 82.0 ± 7.5 days in control mice (Figure 2A). One of 45 control mice and 3 of 42 NaHCO_3_-treated mice did not develop breast tumors within the 9-month observation period starting from cancer induction.

We measure the size of all breast tumors with calipers upon excision 2 weeks after first detection and observe no significant difference in breast tumor burden between control mice and NaHCO_3_-treated mice (Figure 2B). Because oral NaHCO_3_ therapy is not expected to influence our ability to detect tumors by palpation—which is usually possible when tumors are 3–4 mm in diameter [9,49]—tumor burden after 2 weeks is a measure of the tumor growth rate.

The various breast cancer histopathologies occur with similar frequencies in NaHCO_3_-treated mice and control mice (Figure 2C): most numerous are squamous carcinomas and Wnt tumors, whereas adenocarcinomas, adenosquamous carcinomas, solid tumors, myoepitheliomas, solid nodular tumors, and metaplastic carcinomas are less common, consistent with earlier reports [9].

Breast tumor-free survival and breast tumor burden depend on histopathology, but we observe no systematic differences between NaHCO_3_-treated mice and control mice (Figure 2D).

### 2.4. Metabolic Activity Changes during Carcinogenesis and Oral NaHCO_3_ Therapy

**Figure 3:**
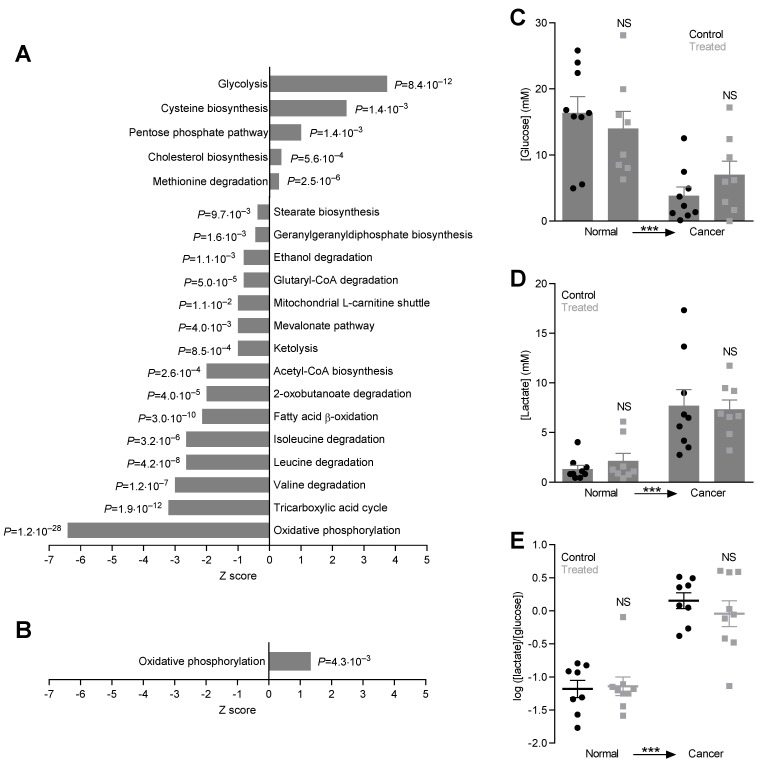
Metabolic pathways are dramatically perturbed during breast carcinogenesis but glycolytic metabolism in breast cancer tissue and normal breast tissue in vivo is unaffected by oral NaHCO_3_ therapy. (**A, B**). Protein expression changes for metabolic pathways. We compare breast cancer tissue vs. normal breast tissue from control mice (**A**) and breast cancer tissue from NaHCO_3_-treated mice vs. control mice (**B**). *p*-values were calculated based on Fisher’s right-tailed exact test. (**C–E**)**.** Interstitial concentrations of glucose (**C**) and lactate (**D**) and corresponding [glucose]/[lactate]-ratios (**E**) measured in microdialysis samples from breast cancer tissue and matched normal breast tissue of NaHCO_3_-treated mice and control mice (*n* = 8–9). Data were compared by repeated measures to-way ANOVA followed by Sidak’s post-tests. NS: not significantly different vs. Control. ***p < 0.001.

Dramatic changes in energy fluxes occur during carcinogenesis as supported by the massively perturbed protein expression profile in breast cancer tissue compared to normal breast tissue (Figure 3A). Most notably, enzymes of the glycolytic pathway increase substantially (Figure 3A and Figure S2A) supporting their dominant contribution to supply of energy and chemical components for cell proliferation in cancer tissue [50]. We also see enrichment of the pentose phosphate pathway (Figure 3A and Figure S2A). In sharp contrast, the majority of enzymes in other—particularly oxidative—catabolic pathways show reduced expression levels as demonstrated for acetyl-CoA biosynthesis, the tricarboxylic acid cycle, and oxidative phosphorylation (Figure 3A and Figure S2A). Reduced or perturbed expression levels are also evident for catabolic pathways involving amino acids (valine, leucine, isoleucine, lysine, and tryptophan), fatty acids (L-carnitine shuttle and fatty acid β-oxidation), ketone bodies, and ethanol (Figure 3A and Figure S2A). These protein expression changes support the Warburg effect, favoring energy production through fermentative glycolysis rather than oxidative phosphorylation. Interestingly, we also see disturbances in cholesterol and stearate biosynthesis (Figure 3A and Figure S2A).

**Figure 4:**
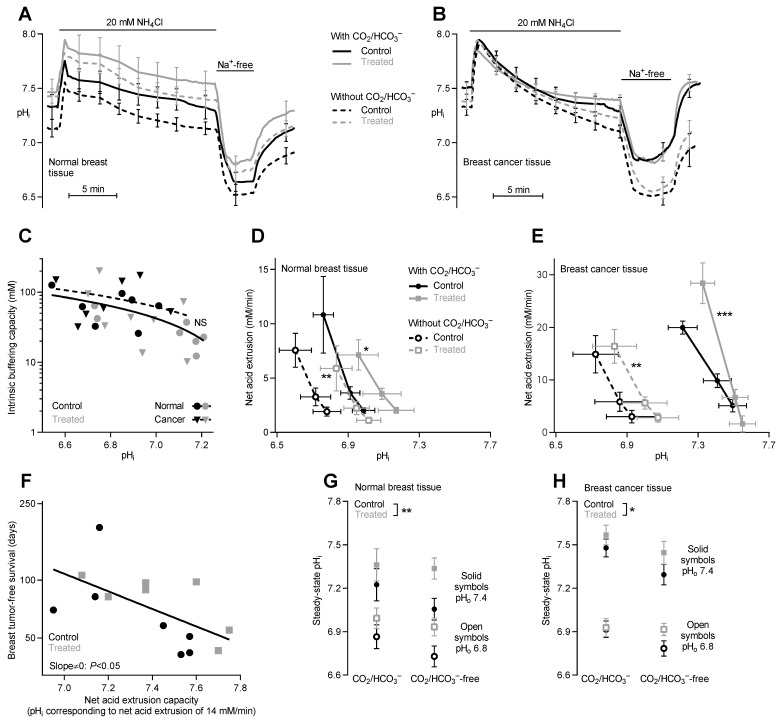
The capacity for net acid extrusion is increased in both normal breast tissue and breast cancer tissue from NaHCO_3_-treated mice compared to control mice. (**A, B**)**.** Average traces of pH_i_-dynamics during NH_4_^+^-prepulse experiments performed on normal breast tissue (**A**) and breast cancer tissue (**B**) from NaHCO_3_-treated mice and control mice (*n* = 7). Experiments were conducted in both the presence and absence of CO_2_/HCO_3_^–^. (**C**)**.** Intracellular intrinsic buffering capacity of organoids of both cancer and normal tissue from NaHCO_3_-treated and untreated mice (*n* = 7). Linear regression showed no significant difference in buffering capacity between NaHCO_3_-treated and control mice. (**D,E**)**.** Cellular net acid extrusion in epithelial organoids isolated from normal breast tissue (**D**) and breast cancer tissue (**E**) displayed as a function of pH_i_. Experiments were based on tissue from NaHCO_3_-treated mice and control mice (*n* = 7) and performed in both the presence and absence of CO_2_/HCO_3_^–^. Data were compared by least-squares linear regression analyses. (**F**). Breast tumor-free survival plotted as function of net acid extrusion capacity displayed as the pH_i_ value corresponding to net acid extrusion of 14 mM/min. The higher the pH_i_ value, the greater the capacity for net acid extrusion. **(G,H).** Steady-state pH_i_ values measured from normal breast tissue (**G**) and breast cancer tissue (**H**) isolated from NaHCO_3_-treated mice and control mice (*n* = 7) and investigated at pH_o_ 7.4 (closed symbols) and 6.8 (open symbols). Data were compared by three-way ANOVA. *p < 0.05, **p < 0.01, ***p < 0.001 vs. Control under similar conditions or as indicated.

Oral NaHCO_3_ therapy partly reverses the downregulation of oxidative phosphorylation in the breast cancer tissue without influencing the upregulation of glycolysis (Figure 3A,B and Figure S2B), and the intervention to neutralize the acidity of the tumor microenvironment thus potentially improves energy supply to the cancer cells. Surprisingly, despite the marked differences in cancer cell pH_i_ (Figure 4) and pH of the tumor microenvironment (Figure 1A), our proteomics analysis reveals no other significant changes of metabolic pathways in the tumor tissue in response to oral NaHCO_3_ therapy.

We sampled interstitial solution by microdialysis in order to functionally evaluate glycolytic metabolism in breast cancer tissue and normal breast tissue in vivo. Consistent with the protein expression patterns (Figure 3A,B), we observe (a) decreased [glucose] (Figure 3C) and increased [lactate] (Figure 3D) in the tumor interstitium as signs of elevated glycolytic activity compared to normal breast tissue and (b) no difference in [lactate]/[glucose]-ratio—which is a measure of glycolytic metabolism—between NaHCO_3_-treated mice and control mice (Figure 3E).

### 2.5. Oral NaHCO_3_ Therapy Increases the Capacity for Net Acid Extrusion in Breast Tissue

Earlier reports demonstrate that Na^+^, HCO_3_^–^-cotransport and Na^+^/H^+^-exchange constitute the major components of net acid extrusion from breast tissue [8,9,10,11]. In accordance, pH_i_ recovery after NH_4_^+^-prepulse-induced acidification is largely Na^+^-dependent in organoids from normal breast tissue and breast cancer tissue irrespective of whether they are isolated from control mice or NaHCO_3_-treated mice (Figure 4A,B).

As expected, the intracellular buffering power of the breast tissue varies as function of pH_i_ (Figure 4C); and consistent with a previous report [9], we find a tendency towards higher intracellular buffering capacity in the breast cancer tissue compared to normal breast tissue although this does not reach statistical significance (Figure 4C). Otherwise, the intracellular buffering capacity is unaffected by oral NaHCO*_3_* therapy.

**Figure 5:**
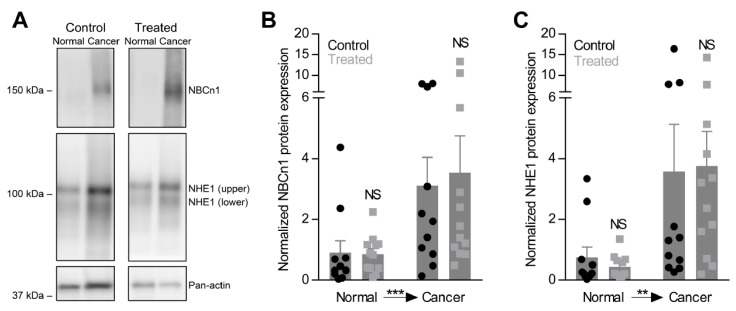
NBCn1 and NHE1 protein expression is increased in organoids from breast cancer tissue compared to normal breast tissue, but unaffected by oral NaHCO_3_ therapy. (**A–C**)**.** Representative immunoblots (**A**) and quantified protein expression levels for NBCn1 (**B**) and NHE1 (**C**) in breast cancer tissue and normal breast tissue from NaHCO_3_-treated mice and control mice (*n* = 11–12). Equal amounts of total protein were loaded in each lane of the gels. The band densities for NBCn1 and NHE1 are normalized to that of pan-actin. Data were compared by repeated measures two-way ANOVA followed by Bonferroni post-tests. **p < 0.01, ***p < 0.001. NS: not significantly different vs. Control. The Full uncropped images of immunoblots please find in Figure S4.

The capacity for net acid extrusion is strongly increased in breast carcinomas compared to normal breast tissue particularly when experiments are performed in presence of CO_2_/HCO_3_^–^ (Figure 4D,E). This finding is consistent with the previously reported key role of NBCn1 for net acid extrusion from breast cancer tissue [9,10] and the upregulated NBCn1 protein expression in breast cancer tissue compared to normal breast tissue (Figure 5A,B). We also observe upregulation of NHE1 protein expression during breast carcinogenesis (Figure 5A,C). Supporting the pathophysiological relevance of local acid-base dynamics for malignancy, we show that the capacity for net acid extrusion correlates negatively with breast tumor-free survival as tumors developing with the shortest latencies show the highest acid extrusion capacities (Figure 4F).

To our surprise, oral NaHCO_3_ therapy increases the capacity for net acid extrusion in breast cancer tissue and normal breast tissue (Figure 4A,B,D,E). The elevated net acid extrusion is evident from the ability of the breast tissue from NaHCO_3_-treated mice to extrude intracellular acid at more alkaline pH_i_ [8,9,10] and is apparent whether organoids are investigated in physiological buffer or are acutely deprived of CO_2_/HCO_3_^−^ (Figure 4D,E). Thus, the data demonstrate that oral NaHCO_3_ therapy increases Na^+^/H^+^-exchange activity and maybe has additional effects on Na^+^, HCO_3_^−^-cotransport. Oral NaHCO_3_ therapy has no additional effect on the upregulation of NBCn1 or NHE1 protein expression during breast carcinogenesis (Figure 5A-C) suggesting that the acid-base transport activity is post-translationally regulated by oral NaHCO_3_ therapy.

### 2.6. Oral NaHCO_3_ Therapy Increases Steady-State pHᵢ in Breast Tissue

Consistent with the increased net acid extrusion capacity, steady-state pHᵢ is significantly elevated in organoids isolated from normal breast tissue (Figure 4G) and breast cancer tissue (Figure 4H) of NaHCO_3_-treated mice compared to control mice irrespective of whether the intracellular acid-base conditions are studied at pH_o_ 7.4 or 6.8.

### 2.7. Oral NaHCO_3_ Therapy Increases ex vivo Proliferation in Organoids

**Figure 6:**
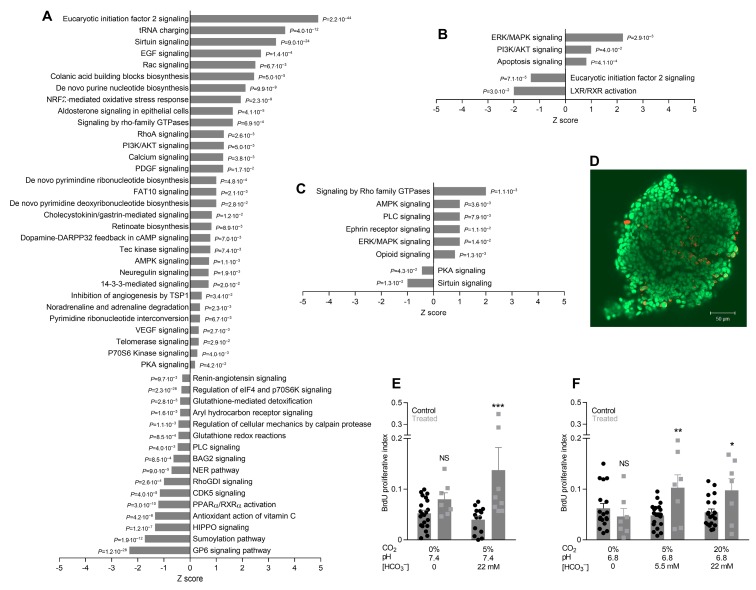
Breast cancer organoids from mice undergoing oral NaHCO_3_ therapy show increased proliferative activity in vitro at both pH_o_ 6.8 and 7.4 but only when investigated in the presence of CO_2_/HCO_3_^–^ buffer. (**A–C**). Protein expression changes for cellular signaling cascades. *p*-values were calculated based on Fisher’s right-tailed exact test. We compare breast cancer tissue vs. normal breast tissue from control mice (**A**), breast cancer tissue from NaHCO_3_-treated mice vs. control mice (**B**), and normal breast tissue from NaHCO_3_-treated mice vs. control mice (**C**). (**D**). Original image of breast cancer organoid fluorescently labeled using an anti-bromodeoxyuridine (BrdU) antibody (red). Nuclei are stained with SYTO16 (green). (**E, F**). BrdU proliferative index in breast cancer organoids from NaHCO_3_-treated mice and control mice (*n* = 7–21) investigated at pH_o_ 7.4 (**E**) and 6.8 (**F**). The BrdU proliferation index is the fraction of cells positive for BrdU after 6 h incubation and represents the rate of cell division during this period. Data were compared by two-way ANOVA followed by Bonferroni post-tests. *p < 0.05, **p < 0.01, ***p < 0.001, NS: not significantly different vs. Control under similar conditions.

Proliferative activity is elevated in breast cancer tissue compared to normal breast tissue [9] and accompanied by increased expression of pathways for nucleotide and protein synthesis (Figure 6A). As shown in Figure 6B,C, we observe mixed consequences of oral NaHCO_3_ therapy, which for some signaling pathways (phosphoinositide 3-kinase (PI3K)/protein kinase B (AKT), Rho family guanosine triphosphatases (GTPases), and 5’ adenosine monophosphate-activated protein kinase (AMPK)) amplify or resemble the protein expression changes seen during breast carcinogenesis (Figure 6A). Other signaling pathways (eukaryotic initiation factor 2, protein kinase A (PKA), phospholispase C (PLC), and Sirtuin) are oppositely affected by oral NaHCO_3_ therapy (Figure 6B,C) and carcinogenesis (Figure 6A).

Cell proliferation is significantly increased in breast cancer organoids isolated from NaHCO_3_-treated mice compared to control mice, and this effect is observed at both pH_o_ 7.4 (Figure 6E) and 6.8 (Figure 6F) but only when experiments are performed in the presence of CO_2_/HCO_3_^–^. These findings show that oral NaHCO_3_ therapy fundamentally influences cancer cell functions and support that the effect of oral NaHCO_3_ therapy depends on local acid-base conditions.

## 3. Discussion

Oral NaHCO_3_ therapy elevates pH of the tumor microenvironment (Figure 1A) but—contrary to our expectation—has no net beneficial effect on breast cancer development or tumor growth (Figure 2). This finding is unlike previous studies showing delayed and reduced onset of prostate cancer development in mice treated with NaHCO_3_ [38]. Consistent with the absence of a net beneficial treatment effect on breast carcinogenesis and early tumor progression, we observe that oral NaHCO_3_ therapy upregulates the cellular capacity for net acid extrusion (Figure 4A–E), which correlates with earlier tumor development (Figure 4F), enhances proliferative activity in breast cancer tissue (Figure 6D–F), and maintains expression of enzymes involved in oxidative phosphorylation (Figure 3A,B). The effects of carcinogenesis and NaHCO_3_ therapy on protein expression are widespread and—in addition to intermediary metabolism (Figure 3)—encompass signaling pathways related to cell-cell and cell-matrix interactions, cytoskeletal dynamics, and immune functions (Figure 1F–H) as well as growth factor signaling, cell proliferation, and apoptosis (Figure 6A–C). The perturbation of granulocyte-macrophage colony-stimulating factor (GM-CSF) in breast cancer tissue (Figure 1F and Figure S1A) is particularly interesting as GM-CSF secretion from orthotopic primary breast tumor models was previously found to depend on carbonic anhydrase activity and lead to recruitment of granulocytic myeloid-derived suppressor cells to pre-metastatic niches in the lung [51]. Based on the consequences of oral NaHCO_3_ therapy, our data suggest that the acidic tumor microenvironment keeps in check some elements of cancer cell function—that are enhanced or reduced compared to normal cells—whereas others are exacerbated.

Upregulation of net acid extrusion is crucial for tumor progression [5,52,53] and associated with poor prognosis [6,54]. The current study demonstrates that elevated net acid extrusion capacity is associated with accelerated breast cancer development (Figure 4F). The enhanced Na^+^/H^+^-exchange activity takes place despite unchanged overall NHE1 protein expression levels as was previously reported in MCF7 breast cancer cells with overexpression of an NH_2_-truncated ErbB2 receptor [55] and in smooth muscle cells with disrupted expression of NBCn1 [56]. Elevated pH_i_ has also been linked to increased protein and DNA synthesis leading to accelerated cell proliferation [57]; and indeed, we observe increased proliferation rates in vitro in breast cancer organoids from NaHCO_3_-treated mice (Figure 6). This could, in part, relate to elevated pH_i_ and NaHCO_3_ therapy (Figure 3, 4) facilitating intermediary metabolism [4]. In human breast cancer tissue, NBCn1 and NHE1 are predominantly co-expressed in cytokeratin-19-positive epithelial cells [11] although limited stromal expression of NBCn1 and NHE1 is consistent with the important function of these transporters in resistance arteries [56,58,59].

Immune cell infiltration and function are among previously reported targets of acidic pH_o_ [17,42]. In congruence, oral administration of NaHCO_3_ has been found to increase T cell infiltration in tumors and potentiate anti-cancer responses during immunotherapy [34]. These effects are consistent with the observed proteomics signs of enhanced chemokine signaling and macrophage activation in the current study (Figure 1G,H). Because we used a model of carcinogen-induced breast cancer in immunocompetent mice, it is surprising that the more neutral tumor pH as result of the oral NaHCO_3_ therapy (Figure 1A) did not cause prominent anti-cancer effects. Differences in the magnitude of the pH_o_-increase attained by oral NaHCO_3_ therapy may determine whether beneficial or detrimental effects dominate the overall treatment outcome. We observe that tumor pH in response to oral NaHCO_3_ therapy increases to around 7.0 in breast carcinomas (Figure 1A) whereas other studies have reported pH_o_ values anywhere between 7.0 and 7.8 [32,34,60].

We previously found that inhibition of net acid extrusion from breast cancer cells by genetic disruption of Na^+^, HCO_3_^–^-cotransporter NBCn1 results in tumors of less malignant phenotype [9,10]. The microenvironmental alkalinisation observed in response to oral NaHCO_3_ therapy does not have a similar effect (Figure 2C). This difference is likely explained by contrasting effects on pH_i_, which is lowered in cancer cells from NBCn1 knockout mice [9,10] but elevated during NaHCO_3_ therapy (Figure 4G,H). The substantial sensitivity of pH_i_ to extracellular acidification also in breast cancer cells (Figure 4G,H) is consistent with previous findings from human and murine breast cancer tissue [8,9,10] and is likely explained by the prominent inhibition of NBCn1 (~60%) and NHE1 (~85%) activity when pH_o_ is lowered to 6.8 [61]. These findings reinforce the suggestion [17] that upregulated net acid extrusion capacity favors malignant functions particularly when cancer cells, for instance during invasion, encounter environments of more neutral pH.

Increased systemic pH could be of concern when orally supplying NaHCO_3_ for cancer therapy [62]. Urinary alkalinization has been observed during increased NaHCO_3_ intake [37] but without significant changes in blood pH [32,37]. Differing from prior studies, we find that oral NaHCO_3_ therapy increases arterial blood pH although the effect is of modest magnitude (~0.04 pH-units, Figure 1B). We collected arterial blood from anesthetized, endotracheally intubated mice that were mechanically ventilated to a fixed expiratory end-tidal CO_2_ fraction whereas other investigators have drawn blood from euthanized mice [37] or used tail bleeds [32]. Although intubating and ventilating mice might eliminate a respiratory compensation of the metabolic alkalosis, it is extremely difficult to obtain reliable blood samples from awake mice without causing stress-induced hyperventilation [63]. With our approach, we sample blood under tightly controlled conditions, which allows us to identify even small changes in acid-base conditions brought about by a metabolic disturbance.

In conclusion, the current study of murine breast carcinogenesis reveals that oral NaHCO_3_ therapy is a double-edged sword that can have both pro- and anti-neoplastic effects. The precise mechanisms modifying cancer development and progression are multifaceted, likely cancer type-dependent, and still not comprehensively understood. Oral NaHCO_3_ therapy increases the capacity for net acid extrusion in breast tissue, which is associated with poor prognosis. We also observe increased proliferation in breast cancer organoids from NaHCO_3_-treated mice and complex changes in protein expression patterns that can facilitate cancer development and progression. Rather than using buffer therapy—with the risk of elevating pH_i_ and promoting cancer development and progression—we propose that the acidic microenvironment of breast cancer tissue can be targeted more successfully based on inhibitors of acid-base transport.

## 4. Materials and Methods

Mice from Janvier Labs (France) were housed in the animal facility at Department of Biomedicine, Aarhus University, under a 12-h light/12-h dark cycle with constant room temperature and humidity. The Danish Animal Experiments Inspectorate approved the animal procedures (2014-15-0201-00330).

### 4.1. Tumor Induction and Oral NaHCO_3_ Therapy

Medroxyprogesterone acetate pellets (50 mg, 90 day release; Innovative Research of America, Sarasota, FL, USA) were implanted subcutaneously in 6 weeks old female C57BL/6j mice that were subsequently treated at 9, 10, 12, and 13 weeks of age with dimethylbenz(α)anthracene through oral gavage [64]. 

At beginning of tumor induction, mice were randomly assigned to receive either water containing 200 mM NaHCO_3_ (treated) or regular water (control) [32,38]. From completion of breast cancer induction, we palpated the mice twice weekly for early tumor detection. Fourteen days after first tumor detection, the mice were anesthetized by intraperitoneal injection of ketamine (80 mg/kg Ketaminol^®^ vet; Intervet International, Boxmeer, Netherlands) and xylazine (8 mg/kg Narcoxyl^®^ vet; Intervet International, Boxmeer, Netherlands) and subjected to microdialysis sampling or tumor pH measurements.

### 4.2. Microdialysis

We measured [glucose] and [lactate] in microdialysates using a CMA 600 Microdialysis Analyzer (CMA Microdialysis AB, Kista, Sweden) [8,9]. Samples were collected by inserting microdialysis probes (CMA 20 Elite, 4 mm membrane length; CMA Microdialysis AB, Kista, Sweden) into breast tumors and matched normal breast tissue guided by needles and split tubing. Dual syringe pumps (Pump 33; Harvard Apparatus, Holliston, MA, USA) perfused the probes at 0.5 µL/min; and after a 60-minute washout period, 6 µL sample was collected for analysis.

### 4.3. Tumor pH

Anaesthetized mice were endotracheally intubated and kept on a ventilator (Minivent type 845, Hugo Sachs Electronic, March, Germany). The expiratory end-tidal CO_2_ fraction was measured by capnography (Capnograph type 340; Hugo Sachs Electronic, March, Germany) and maintained at ~3.8% throughout the experiments. Glass pH microelectrodes (pH 500; Unisense, Aarhus, Denmark) were advanced stepwise into the tumors 2 mm at a time, and the reference electrode placed in the intraperitoneal space. We observed pH decreases as the electrode tip advanced deeper into the tumor and pH increases when the electrode moved towards more superficial tumor regions. Figure 1A displays the lowest pH for each tumor. We measured intraperitoneal pH at the end of each experiment and included mice where intraperitoneal pH was between 7.1 and 7.7.

### 4.4. Tumor Size and Histopathology

Tumors were removed postmortem and measured in 3 perpendicular dimensions (*s*, *m*, *l*) with calipers. Tumors were considered ellipsoid, and their volume (*V*) calculated as *V*=*s*·*m*·*l*·π/6.

Tissue samples fixed in formalin for 30 min (normal tissue) or 1 h (cancer tissue) were stored in phosphate-buffered saline, paraffin-embedded, cut to 3-µm sections, and stained with hematoxylin and eosin. An experienced breast pathologist evaluated histopathology according to previous studies [9].

### 4.5. Organoid Isolation

Finely chopped excised breast cancer tissue and matched normal breast tissue incubated for 4 hours on a shaking table (set at 60 rpm) at 37 °C in advanced DMEM/F12 culture medium (Life Technologies, Nærum, Denmark) aerated with 5% CO_2_/balance air and supplemented with 10% fetal bovine serum (Biochrom, Cambridge, UK), 1% Glutamax (Gibco, Invitrogen, Roskilde, Denmark), and 450 IU/mL collagenase type 3 (Worthington Biochemical Corporation, Lakewood, NJ, USA). Organoids are 100–150 µm in diameter and consist primarily of cytokeratin-19-positive cancer cells with smaller numbers of other cell types (e.g., myofibroblasts and tumor-associated macrophages) [8,9]. From the partially digested breast tissue, organoids were isolated by sedimentation for 20 min and used directly for experiments without culture in order to avoid changes in expression profile or phenotype.

### 4.6. Intracellular pH

We performed fluorescence-based pH_i_ measurements on freshly isolated breast epithelial organoids using a Diaphot 200 wide-field microscope (Nikon, Tokyo, Japan) equipped with an R1 or SRV CCD Retiga fluorescence camera (QImaging, Surrey, Canada) controlled through VisiView software (Visitron Systems, Puchheim, Germany). Organoids maintained at 37 °C were loaded with 3 µM BCECF-AM (Invitrogen, Roskilde, Denmark) in 0.1% DMSO for 20 min. During experiments performed with CO_2_/HCO_3_^–^ present, the bath solution was continuously bubbled with 5% CO_2_/balance air. Organoids were excited alternatingly at 495 and 440 nm and emission light collected at 510 nm. Fluorescence ratios were calibrated to pH based on the high [K^+^]-nigericin technique [65]. Net acid-base transport activity was determined from the pH_i_ recovery rate after NH_4_^+^-prepulse-induced intracellular acidification [8,9,66]. Intrinsic buffering capacity calculated from NH_4_Cl-induced changes in pH_i_ under CO_2_/HCO_3_^–^-free conditions did not significantly differ between NaHCO_3_-treated mice and control mice or between organoids from breast cancer tissue and normal breast tissue (Figure 4C). We quantified the intracellular acid load using the Henderson-Hasselbalch equation, assuming equilibrium of NH_3_ across cell membranes. Contribution of CO_2_/HCO_3_^–^ to intracellular buffering capacity was calculated from *β* = 2.3·[HCO_3_^–^]_i_ [58,67].

Physiological saline solutions for functional experiments consisted of (in mM): 4 K^+^, 140 Na^+^, 1.6 Ca^2+^, 1.2 Mg^2+^, 122 Cl^–^, 1.18 H_2_PO_4_^–^, 22 HCO_3_^−^, 10 HEPES, 5.5 glucose, 0.03 EDTA, 1.2 SO_4_^2–^, aerated with 5% CO_2_/balance air, adjusted to pH 7.4 at 37 °C. In experiments performed at pH_o_ 6.8, the concentration of HCO_3_^–^ was reduced to 5.5 mM through substitution with Cl^–^. In Na^+^-free solutions, *N*-methyl-D-glucammonium replaced Na^+^, except for NaHCO_3_ that was replaced with choline-HCO_3_. All solutions contained 5 mM probenecid in order to inhibit BCECF extrusion by the organic anion transporters [8,9].

### 4.7. Cell Proliferation

Freshly isolated organoids from breast cancer tissue incubated 6 hours with 0.1% bromodeoxyuridine (BrdU, Invitrogen, Roskilde, Denmark) at 37 °C. We performed experiments with CO_2_/HCO_3_^–^ under control circumstances (pH 7.4, 5% CO_2_, 22 mM HCO_3_^–^), respiratory acidosis (pH 6.8, 20% CO_2_, 22 mM HCO_3_^–^), and metabolic acidosis (pH 6.8, 5% CO_2_, 5.5 mM HCO_3_^–^). Experiments without CO_2_/HCO_3_^–^ were performed at pH 7.4 and 6.8. All solutions contained 10 mM HEPES. After incubation, organoids were fixed in 75% ethanol for 2 min and then denatured in 1 M HCl. We identified dividing cells by immunofluorescence imaging using anti-BrdU mouse monoclonal primary antibody (#5292, Cell Signaling Technology, Danvers, MA, USA), Alexa488-labelled donkey anti-mouse secondary antibody (#A21202, Invitrogen, Roskilde, Denmark), and a LSM510META confocal laser scanning microscope (Zeiss, Oberkochen, Germany) [68]. Nuclei were co-stained with SYTO16 (Life Technologies, Nærum, Denmark). We manually calculated proliferation indices (BrdU-positive cells/SYTO16-positive nuclei) for 3 different focal planes in each organoid using ImageJ software (NIH, Bethesda, MD, USA).

### 4.8. Immunoblotting

Organoids were homogenized in a lysis buffer (20 mM Tris-HCl, 150 mM NaCl, 5 mM EGTA (pH 7.5), 10 mM NaF, 20 mM sodium β-glycerophosphate, and HALT protease and phosphatase inhibitor cocktail (Thermo Scientific, Waltham, MA, USA)) using pellet pestles (Sigma-Aldrich, St. Louis, MO, USA), sonicated for 45 s, and centrifuged at ~16,000 g for 10 min at 4 °C. We measured total protein concentrations in the supernatants based on a bicinchoninic acid protein assay kit (Thermo Scientific, Waltham, MA, USA). 10 µg total protein diluted in Laemmlie sample buffer was loaded in each lane of a sodium dodecyl sulfate polyacrylamide gel (Bio-Rad, Hercules, CA, USA), separated by gel electrophoresis, and transferred to polyvinylidene difluoride membranes blocked with 0.3% i-block (Applied Biosystems, Foster City, CA, USA). Although boiling can limit protein aggregation and improve solubility—that may otherwise lead to smears—we did not heat the lysates as we find that this markedly reduces the ability of the antibodies to recognize their specific epitopes. Membranes were probed with rabbit anti-NBCn1 (1:200; kind gift from Dr. Jeppe Praetorius, Aarhus University, Aarhus, Denmark) [69] or mouse monoclonal anti-NHE1 (1:500; #sc-136239, Santa Cruz Biotechnology, Dallas, TX, USA) [9] primary antibody, and then with species-matched goat anti-rabbit (1:2,000; #7074, Cell Signaling Technology, Danvers, MA, USA) or horse anti-mouse (1:2,000; #7076, Cell Signaling Technology, Danvers, MA, USA) secondary antibody conjugated to horseradish peroxidase. Bound antibody was detected by enhanced chemiluminescence (ECL Plus; GE Healthcare, Chicago, IL, USA) and quantified using Image Studio Lite version 5.2 (LI-COR Biosciences, Lincoln, NE, USA). NBCn1 and NHE1 expression was normalized to pan-actin expression.

### 4.9. Label-Free Quantitative Nano Liquid Chromatography-Tandem Mass Spectrometry (LFQ nLC-MS/MS)

Organoids from Wnt type breast cancer tissue and matched normal breast tissue were snap frozen in liquid N_2_, stored at –80 °C, homogenized in lysis buffer (5% sodium deoxycholate, 20 mM triethylammonium bicarbonate), and sonicated on ice [70]. Protein concentrations were measured by IR spectrometry (Direct Detect Spectrometer, Merck, Kenilworth, NJ, USA). Up to 100-µg protein was reduced, alkylated, and digested using filter-aided preparation (Microcon 30K centrifugal filter device, Merck, Kenilworth, NJ, USA). Peptide concentrations were measured by tryptophan fluorescence [70], and 1 µg peptide injected from each sample. Two of 28 samples from 7 NaHCO_3_-treated mice and 7 control mice could not be run due to irregularities with chromatographic peptide separation.

Peptide mixtures were separated by nano Liquid-Chromatography (Ultimate 3000; Thermo Scientific, Waltham, MA, USA) coupled to an Orbitrap Fusion Tribrid mass spectrometer through an EASY-Spray nano-electrospray ion source (Thermo Scientific, Waltham, MA, USA). A µ-Precolumn (300 µm × 5 mm, C18 PepMap100, 5 µm, 100 Å; Thermo Scientific, Waltham, MA, USA) and analytical column (EASY-Spray Column, 750 mm × 75 µm, PepMap RSCL, C18, 2 mm, 100 Å; Thermo Scientific, Waltham, MA, USA) trapped and separated peptides, respectively. Peptides were eluted with a flow of 300 nL/min. The elution gradient was made by mixing a buffer containing 99.9% water and 0.1% formic acid with a buffer containing 80% acetonitrile, 20% water, and 0.1% formic acid. The universal method setting was used for mass spectrometry detection with full Orbitrap scans (m/z 400–1500) at a resolution of 120,000, an automatic gain control (AGC) target of 4·10^5^, a maximum injection time of 50 ms, and a cycle time of 3 s. The most intense precursors were selected with an intensity threshold of 5·10^3^. Charge states 2–7 were included. MS^2^ scans were performed in the linear ion trap at rapid scan rate with collision-induced dissociation energy at 35%, an AGC target of 2·10^3^, and a maximum injection time of 300 ms. The precursor ions were isolated using the quadrupole set with an isolation window of 1.6 m/z. Dynamic exclusion was set to 60 s. Internal mass calibration was used by activating the Easy-IC using fluoranthene.

The 26 raw data files were used to search the Mus musculus database from Uniprot downloaded on 21 March 2018 using MaxQuant (version 1.5.5.1) for LFQ analysis [71]. Carbamidomethyl (C) was used as fixed modification. The false discovery rate for PSM, protein identification hits, and proteins identified by site were each set at 1%. The LFQ minimum ratio count was set to 1. MS/MS was required for LFQ comparisons. Unique and razor peptides, unmodified and modified with oxidation (M) or acetyl (protein *N*-terminal), were used for protein quantification with a minimum ratio count of 2. The match between runs function was used. Revert sequences were used for decoy search. Contaminant sequences were included in the search. The generated results file was then entered into Perseus (version 1.6.1.1) [72] where data were log2 transformed and filtered (at least 2 unique peptides for identification in at least 70% of samples in each group). This approach identified ~2500 proteins (Table S1) that we analyzed using Ingenuity Pathway Analysis software (version 49932394, Qiagen, Hilden, Germany).

### 4.10. Blood and Urine

We treated C57BL/6j mice with oral NaHCO_3_ for 151 days (average treatment duration for mice undergoing cancer induction) and collected spontaneously released urine on Parafilm^®^. We then anaesthetized and mechanically ventilated mice to normocapnia (expiratory end-tidal CO_2_ fraction of ~3.8%), as described above, for 10 min prior to blood sampling by carotid artery puncture. We analyzed urine and blood immediately after collection with an ABL80 FLEX (Radiometer, Brønshøj, Denmark). We excluded a few blood samples due to signs of hemolysis ([K⁺] > 6 mM) or inadequate ventilation (pO_2_ ˂ 80 mmHg and/or pCO_2_ > 60 mmHg).

### 4.11. Statistics

Data are expressed as mean ± SEM. *p* ˂ 0.05 is considered statistically significant; *n* equals number of mice, except in proliferation experiments where n equals number of organoids. We compared one variable between two normally distributed groups of equal variance using two-tailed Student’s *t*-tests (paired or unpaired, as appropriate). For 2 groups of unequal variance, we used *t*-tests with Welch’s correction; and for data that are not normally distributed, we used non-parametric Mann-Whitney tests. We compared one variable between more than 2 groups using repeated measures one-way ANOVA followed by Sidak’s post-tests. We tested effects of 2 variables on a third variable using repeated measures two-way ANOVA followed by Bonferroni post-tests and effects of 3 variables on a fourth variable using three-way ANOVA. We compared Kaplan-Maier curves by Gehan-Breslow-Wilcoxon test. We assessed linear relationships based on least-squares regression analyses. Right-skewed data were log-transformed before analyses to obtain normal distribution. Analyses were performed using Prism 7.03 (GraphPad, San Diego, CA, USA) except for mass spectrometry data analyzed by Fisher’s right-tailed exact test in Ingenuity Pathway Analysis software (Qiagen, Hilden, Germany). 

## 5. Conclusions

Oral NaHCO_3_ therapy neutralizes the microenvironment of breast carcinomas, elevates the capacity for cellular net acid extrusion, and accelerates proliferation without net effect on breast cancer development or tumor growth. These unexpected pro-neoplastic consequences of oral NaHCO_3_ therapy in breast tissue cancel out previously reported anti-neoplastic effects. 

## Supplementary Materials

The following are available online at https://www.mdpi.com/2072-6694/12/4/891/s1, Figure S1–S3: Illustrated results of Ingenuity Pathway Analyses, Figure S4: Full uncropped images of immunoblots from Figure 5, Table S1: Mass spectrometry data.
