# Supplementary material for: Targeting the Acidic Tumor Microenvironment: Unexpected Pro-Neoplastic Effects of Oral NaHCO3 Therapy in Murine Breast Tissue
Source: Cancers (Basel). 2020 Apr 6;12(4):891. doi: 10.3390/cancers12040891 (PMC7226235; doi:10.3390/cancers12040891)
Supplement: Supplementary file 1 [file cancers-12-00891-s001.zip › Supplementary Figures 1-4.docx]

Supplementary Figures for

**Targeting the acidic tumor microenvironment: Unexpected
pro-neoplastic effects of oral NaHCO_3_ therapy in murine breast tissue**

Ninna C. S. Voss^1,2^, Thomas Dreyer^1^, Mikkel B. Henningsen^1^, Pernille Vahl^3^,
Bent Honoré^1^, Ebbe Boedtkjer^1^

^1^Department of Biomedicine, Aarhus University, ^2^Department of Surgery, Regionshospitalet Randers, and ^3^Department of Pathology, Aarhus University Hospital, Denmark

Protein not detected in dataset

Protein with no significant change in expression

Red: Protein with upregulated expression.
Green: Protein with downregulated expression.

**Figure 1A: Breast cancer tissue *vs.* normal breast tissue of Control mice**

I: Interleukin-22 signaling pathway (Z-score: 2, *P*=3.8·10^–2^)

II: Integrin-linked kinase signaling pathway (Z-score: 1.4, *P*=3.7·10^–5^)

III: Regulatory signaling pathway of actin-based motility by Rho (Z-score: 1.291, *P*=1.4·10^–4^)

IV: CD28 signaling pathway in T helper cells (Z-score: 1.265, *P*=2.8·10^–2^)

V: Actin nucleation by ARP-WASP complex pathway (Z-score: 1.155, *P*=2.5·10^–5^)

VI: fMLP signaling pathway in neutrophils (Z-score: 1.155, *P*=2.2·10^–2^)

VII: Pathway for remodeling of epithelial adherence junctions (Z-score: 1.134, *P*=3.3·10^–10^)

VIII: Fcγ receptor-mediated phagocytosis pathway in macrophages (Z-score: 1.069, *P*=4.9·10^–4^)

IX: Actin cytoskeleton signaling pathway (Z-score: 1.043, *P*=3.3·10^–4^)

X: Leukocyte extravasation signaling pathway (Z-score: 0.853, *P*=6.7·10^–5^)

XI: Integrin signaling pathway (Z-score: 0.756, *P*=4.5·10^–6^)

XII: Paxillin signaling pathway (Z-score: 0.535, *P*=7.2·10^–5^)

XIII: GM-CSF signaling pathway (Z-score: 0.378, *P*=3.4·10^–2^)

XIV: Calcium-induced T-lymphocyte apoptosis pathway (Z-score: –0.632, *P*=7.4·10^–4^)

**Figure 1B: Breast cancer tissue of NaHCO_3_-treated mice *vs.* Control mice**

I: Integrin signaling pathway (Z-score: 3, *P*=2.6·10^–4^)

II: Actin cytoskeleton signaling pathway (Z-score: 2.333, *P*=3.0·10^–4^)

III: Paxillin signaling pathway (Z-score: 1.633, *P*=6.9·10^–4^)

IV: Fcγ receptor-mediated phagocytosis pathway in macrophages (Z-score: 1, *P*=1.3·10^–2^)

V: Integrin-linked kinase signaling pathway (Z-score: 0.447, *P*=3.9·10^–2^)

VI: Acute phase response signaling pathway (Z-score: –0.447, *P*=1.9·10^–3^)

**Figure 1C: Normal breast tissue of NaHCO_3_-treated mice *vs.* Control mice**

I: Pathway for NO and ROS production in macrophages (Z-score: 2.236, *P*=2.9·10^–4^)

II: Interleukin-8 signaling pathway (Z-score: 1.633, *P*=4.0·10^–4^)

III: CXCR4 signaling pathway (Z-score: 1, *P*=1.3·10^–3^)

IV: fMLP signaling pathway in neutrophils (Z-score: 1, *P*=2.4·10^–3^)

V: Integrin signaling pathway (Z-score: 0.816, *P*=5.8·10^–4^)

**Figure 2A: Breast cancer tissue *vs.* normal breast tissue of Control mice**

I: Glycolysis (Z-score: 3.742, *P*=8.4·10^–12^)

II: Cysteine biosynthesis (Z-score: 2.449, *P*=1.4·10^–3^)

III: Pentose phosphate pathway (Z-score: 1, *P*=1.4·10^–3^)

IV: Cholesterol biosynthesis (Z-score: 0.378, *P*=5.6·10^–4^)

V: Methionine degradation (Z-score: 0.302, *P*=2.5·10^–6^)

VI: Stearate synthesis (Z-score: –0.378, *P*=9.7·10^–3^)

VII: Geranylgeranyldiphosphate biosynthesis (Z-score: –0.447, *P*=1.6·10^–3^)

VIII: Ethanol degradation (Z-score: –0.816, *P*=1.1·10^–3^)

IX: Glutaryl-CoA degradation (Z-score: –0.816, *P*=5.0·10^–5^)

X: Mitochondrial L-carnitine shuttle (Z-score: –1, *P*=1.1·10^–2^)

XI: Mevalonate pathway (Z-score: –1, *P*=4.0·10^–3^)

XII: Ketolysis pathway (Z-score: –1, *P*=8.5·10^–4^)

XIII: Acetyl-CoA biosynthesis (Z-score: –2, *P*=2.6·10^–4^)

XIV: 2-oxobutanoate degradation pathway (Z-score: –2, *P*=4.0·10^–5^)

XV: Fatty acid β-oxidation (Z-score: –2.138, *P*=3.0·10^–10^)

XVI: Isoleucine degradation pathway (Z-score: –2.646, *P*=3.2·10^–6^)

XVII: Leucine degradation pathway (Z-score: –2.646, *P*=4.2·10^–8^)

XVIII: Valine degradation pathway (Z-score: –3, *P*=1.2·10^–7^)

XIX: Tricarboxylic acid cycle (Z-score: –3.207, *P*=1.9·10^–12^)

XX: Oxidative phosphorylation (Z-score: –6.41, *P*=1.2·10^–28^)

**Figure 2B: Breast cancer tissue of NaHCO_3_-treated mice *vs.* Control mice**

I: Oxidative phosphorylation (Z-score: 1.342, *P*=4.3·10^–3^)

**Figure 3A: Breast cancer tissue *vs.* normal breast tissue of Control mice**

I: Eucaryotic initiation factor 2 signaling (Z-score: 4.902, *P*=2.2·10^–44^)

II: tRNA charging pathway (Z-score: 3.638, *P*=4.0·10^–12^)

III: Sirtuin signaling (Z-score: 3.286, *P*=9.0·10^–24^)

IV: EGF signaling (Z-score: 2.714, *P*=1.4·10^–4^)

V: Rac signaling (Z-score: 2.496, *P*=6.7·10^–3^)

VI: Colanic acid building blocks biosynthesis (Z-score: 2.449, *P*=5.0·10^–5^)

VII: De novo purine nucleotide biosynthesis (Z-score: 2.121, *P*=9.9·10^–9^)

VIII: NRF2-mediated oxidative stress response (Z-score: 1.941, *P*=2.3·10^–9^)

IX: Aldosterone signaling in epithelial cells (Z-score: 1.633, *P*=4.1·10^–5^)

X: Signaling by Rho-family GTPases (Z-score: 1.633, *P*=6.9·10^–4^)

XI: RhoA signaling (Z-score: 1.291, *P*=2.6·10^–3^)

XII: PI3K/AKT signaling (Z-score: 1.291, *P*=5.0·10^–3^)

XIII: Calcium signaling (Z-score: 1.265, *P*=3.8·10^–3^)

XIV: PDGF signaling (Z-score: 1.265, *P*=1.7·10^–2^)

XV: De novo pyrimidine ribonucleotide biosynthesis (Z-score: 1, *P*=4.8·10^–4^)

XVI: FAT10 signaling (Z-score: 1, *P*=2.1·10^–3^)

XVII: De novo pyrimidine deoxyribonucleotide biosynthesis (Z-score: 1, *P*=2.8·10^–2^)

XVIII: Cholecystokinin/gastrin-mediated signaling (Z-score: 0.832, *P*=1.2·10^–2^)

XIX: Retinoate biosynthesis (Z-score: 0.816, *P*=1.2·10^–2^)

XX: Dopamine-DARPP32 feedback in cAMP signaling (Z-score: 0.775, *P*=7.0·10^–3^)

XXI: Tec kinase signaling (Z-score: 0.775, *P*=7.4·10^–3^)

XXII: AMPK signaling (Z-score: 0.728, *P*=1.1·10^–3^)

XXIII: Neuregulin signaling (Z-score: 0.707, *P*=1.9·10^–3^)

XXIV: 14-3-3-mediated signaling (Z-score: 0.707, *P*=2.0·10^–2^)

XXV: Inhibition of angiogenesis by TSP1 (Z-score: 0.447, *P*=3.4·10^–2^)

XXVI: Noradrenaline and adrenaline degradation (Z-score: 0.378, *P*=2.3·10^–3^)

XXVII: Pyrimidine ribonucleotide interconversion (Z-score: 0.378, *P*=6.7·10^–3^)

XXVIII: VEGF signaling (Z-score: 0.333, *P*=2.7·10^–3^)

XXIX: Telomerase signaling (Z-score: 0.333, *P*=2.9·10^–2^)

XXX: P70S6 kinase signaling (Z-score: 0.277, *P*=4.0·10^–3^)

XXXI: Protein kinase A signaling (Z-score: 0.192, *P*=4.2·10^–2^)

XXXII: Renin-angiotensin signaling (Z-score: –0.302, *P*=2.5·10^–2^)

XXXIII: Regulation of eukaryotic initiation factor 4 & P70S6 kinase (Z-score: –0.333, *P*=2.3·10^–28^)

XXXIV: Glutathione-mediated detoxification (Z-score: –0.378, *P*=1.3·10^–3^)

XXXV: Aryl hydrocarbon receptor signaling (Z-score: –0.378, *P*=8.4·10^–5^)

XXXVI: Regulation of cellular mechanics by calpain protease (Z-score: –0.447, *P*=2.5·10^–2^)

XXXVII: Glutathione redox reactions (Z-score: –0.447, *P*=8.1·10^–3^)

XXXVIII: Phospholipase C signaling (Z-score: –0.471, *P*=3.8·10^–2^)

XXXIX: BAG2 signaling (Z-score: –0.632, *P*=7.4·10^–5^)

XXXX: NER pathway (Z-score: –0.707, *P*=7.4·10^–5^)

XXXXI: RhoGDI signaling (Z-score: –1, *P*=8.6·10^–3^)

XXXXII: CDK5 signaling (Z-score: –1.155, *P*=2.0·10^–3^)

XXXXIII: PPARα/RXRα activation pathway (Z-score: –1.213, *P*=2.4·10^–4^)

XXXXIV: Antioxidant action of vitamin C (Z-score: –1.342, *P*=2.9·10^–2^)

XXXV: HIPPO signaling (Z-score: –1.342, *P*=5.8·10^–3^)

XXXXVI: Sumoylation pathway (Z-score: –1.732, *P*=3.4·10^–5^)

XXXXVII: GP6 signaling (Z-score: –2.324, *P*=1.8·10^–3^)

**Figure 3B: Breast cancer tissue of NaHCO_3_-treated mice *vs.* Control mice**

I: ERK/MAPK signaling (Z-score: 2.236, *P*=2.9·10^–3^)

II: PI3K/AKT signaling (Z-score: 1, *P*=4.0·10^–2^)

III: Apoptosis signaling (Z-score: 0.816, *P*=4.1·10^–4^)

IV: Eucaryotic initiation factor 2 signaling (Z-score: –1.342, *P*=7.1·10^–5^)

V: LXR/RXR activation pathway (Z-score: –2, *P*=3.0·10^–2^)

**Figure 3C: Normal breast tissue of NaHCO_3_-treated mice *vs.* Control mice**

I: Signaling by Rho family GTPases (Z-score: 2, *P*=2.9·10^–4^)

II: AMPK signaling (Z-score: 1, *P*=3.6·10^–3^)

III: Phospholipase C signaling (Z-score: 1, *P*=7.9·10^–3^)

IV: Ephrin receptor signaling (Z-score: 1, *P*=1.1·10^–2^)

V: ERK/MAPK signaling (Z-score: 1, *P*=1.4·10^–2^)

VI: Opioid signaling (Z-score: 0.816, *P*=1.3·10^–3^)

VII: Protein kinase A signaling (Z-score: –0.447, *P*=4.3·10^–2^)

VIII: Sirtuin signaling (Z-score: –1, *P*=1.3·10^–2^)

**Figure 4: Full uncropped immunoblot corresponding to Figure 5 in the main paper**
